# MRI-Based Habitat Radiomics for Differentiating Early-Stage Endometrial Carcinoma from Submucous Leiomyoma: A Multicenter Validation Study

**DOI:** 10.7150/jca.129931

**Published:** 2026-05-18

**Authors:** Hao Tian, Anqi Yan, Lei Cao, Ziyi Pan, Yuyan Guo, Bo Shen, Jing Wang, Jingxuan Jiang

**Affiliations:** 1 Department of Radiology, Affiliated Hospital of Nantong University, Nantong, China.; 2 Department of Gynecology, Obstetrics and Gynecology Hospital of Fudan University, Shanghai, China.; 3 Department of Radiology, Haimeng District Traditional Chinese Medicine Hospital, Nantong, China.; 4 Department of Radiology, Affiliated Hospital of Hebei University, Hebei, China.

**Keywords:** endometrial carcinoma, submucous leiomyoma, magnetic resonance imaging, radiomics, habitat

## Abstract

**Objective:**

This study aimed to investigate the utility of magnetic resonance imaging (MRI)-based habitat radiomics for preoperatively distinguishing early-stage endometrial carcinoma (EC) from submucous leiomyoma (SML).

**Materials and Methods:**

A retrospective study was conducted on uterine lesions patients who underwent MRI from three hospitals. The k-means clustering algorithm was applied to segment the MRI into distinct habitats based on T1-weighted imaging (T1WI), T2-weighted imaging (T2WI), and apparent diffusion coefficient (ADC) maps. Radiomic features were extracted from whole-tumor and these habitats and selected by the Pearson correlation coefficient and least absolute shrinkage and selection operator (LASSO) regression. A logistic regression (LR) model was constructed by these radiomics in the training set.

**Results:**

A total of 231 eligible patients were incorporated, 97 EC and 134 SML confirmed by histopathology. In the training cohort, the AUCs of the models based on features from the whole-tumor, habitat_1, habitat_2, and habitat_3 were 0.826, 0.787, 0.770, and 0.907, respectively, while in the test and external validation cohorts, the corresponding AUCs were 0.774/0.751, 0.486/0.608, 0.663/0.514, and 0.858/0.881. Compared with whole-tumor model, habitat_3 model demonstrated incrementally improved predictive performance in the external validation cohort (0.881 [95% CI: 0.799-0.934]).

**Conclusion:**

MRI-based habitat radiomics offers incremental improvement for preoperative differentiation between early-stage EC and SML.

## Introduction

Endometrial carcinoma (EC) stands as a significant gynecological malignancy, posing substantial health challenges due to its increasing incidence and the complexity of distinguishing it from submucous leiomyoma (SML) 1. Accurate preoperative diagnosis is essential for determining the most effective treatment strategies and improving patient outcomes 2-4. While apparent diffusion coefficient (ADC) obtained from magnetic resonance imaging (MRI) is a well-established imaging modality for evaluating uterine pathologies, differentiating early-stage EC from SML can be challenging due to overlapping imaging features, particularly in ADC values. Early-stage EC may exhibit less pronounced diffusion restriction (higher ADC) in well-differentiated tumors, while degenerated SMLs (e.g., with necrosis or edema) can paradoxically show reduced ADC values, mimicking malignancy 5. The advent of radiomics, an emerging field that extracts a high-throughput quantitative imaging phenotype, offers an approach to enhance diagnostic precision 6.

The utility of MRI-based radiomics lies in its ability to quantify the complex imaging features of tumors, capturing the spatial heterogeneity inherent within lesions 7. This method has the potential to reveal subtle differences that may not be apparent through visual inspection alone. Radiomics can provide a comprehensive characterization of the tumor microenvironment 8. The application of advanced analytical techniques, such as the k-means clustering algorithm, allows for the identification of distinct intratumoral habitats that may correspond to different biological behaviors 9.

Despite the growing body of research in radiomics, there is a need to validate its effectiveness in the specific context of EC and SML. This study aims to fill this gap by investigating the potential of MRI-based habitat radiomics to differentiate EC from SML preoperatively. We hypothesize that the integration of radiomic features extracted from distinct MRI sequences will enhance the diagnostic accuracy, offering a non-invasive and robust tool for clinical decision-making.

The significance of this study lies in its potential to transform the diagnostic landscape of endometrial pathologies. By focusing solely on imaging features, this research aims to demonstrate the intrinsic value of radiomics in distinguishing between malignant and benign conditions. The findings may pave the way for personalized medicine approaches, where treatment plans are tailored based on the unique radiomic signature of a patient's tumor.

## Materials and Methods

### Patient Population

**Figure 1:**
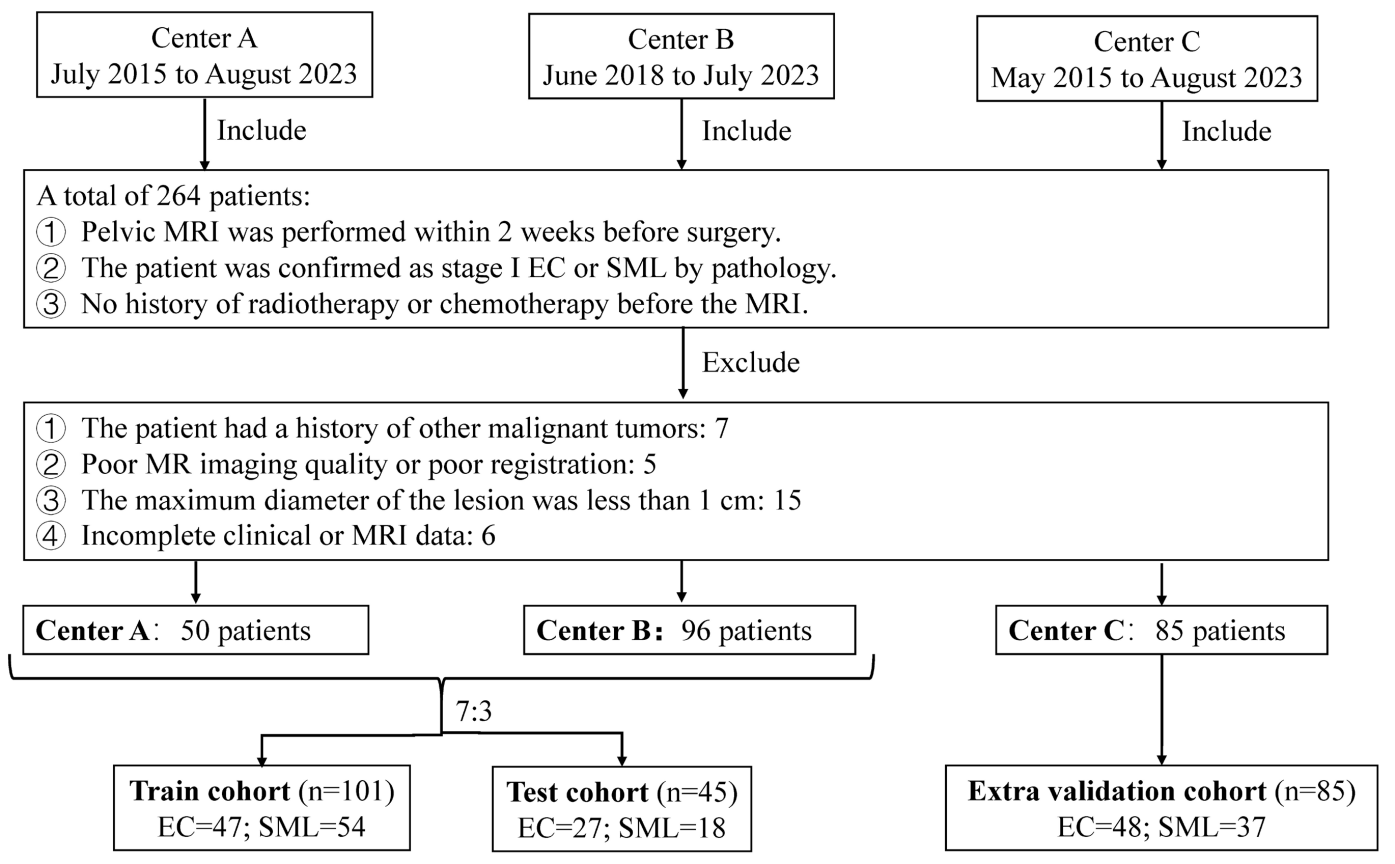
Flowchart of the patient collection. EC: endometrial carcinoma; SML: submucous leiomyoma.

Ethical approval for this retrospective study was obtained from the Ethics Committee of the Haimeng District Traditional Chinese Medicine Hospital (No. KY202412). The study adhered to the ethical guidelines of the 1964 Helsinki Declaration and its subsequent amendments or similar ethical standards. Given the retrospective nature of the study, the requirement for informed consent was waived. Between May 2015 and August 2023, we retrospectively collected 231 patients with uterine lesions from three hospitals (center A, B and C). The inclusion criteria are: (1) pelvic MRI within 2 weeks before surgery; (2) pathologically confirmed stage I endometrial carcinoma (2023 FIGO staging, stages IA-IC) or submucous leiomyoma^10^; (3) no prior radiotherapy or chemotherapy. The exclusion criteria are: (1) history of other malignant tumors; (2) poor MR imaging quality or registration failure; (3) maximum lesion diameter < 1 cm; (4) incomplete clinical or MRI data. The demographic and clinical characteristics of the patients were carefully documented, including age, body mass index (BMI), history of hypertension and diabetes mellitus. The patients from the center A and B were randomly allocated into a training cohort and a test cohort in a 7:3 ratio, the patients from the center C were allocated into an external validation cohort. The flowchart of the patient collection is shown in** Figure 1**.

### MRI Protocols

MRI was performed using 1.5 or 3.0-T scanners (Philips Elition, uMR 770, GE SIGNA, and Siemens Erlangen). A standardized pelvic protocol included the acquisition of T1-weighted imaging (T1WI), T2-weighted images (T2WI), and diffusion-weighted imaging (DWI) with a b-value of 1000s/mm^2^. N4 bias field correction to reduce the non-uniformity of the magnetic field across different scanners. Resampling of isotropic voxels (1×1×1 mm) using B-spline interpolation to address voxel inconsistencies among various sequences. Using ITK-SNAP software (http:// www. itk-snap. org), regions of interest (ROIs) were manually drawn along the tumor margin on each T2WI slice by radiologist 1 (with 3 years of experience in gynecological imaging) and automatically matched to axial T1WI and DWI sequences. After one month, 30 patients were randomly selected for ROI delineation by radiologist 1 and radiologist 2 (with 10 years of experience in gynecological imaging), respectively. The intraclass correlation coefficients (ICCs) were used to assess the reproducibility of radiomics features.

### Habitat Imaging

The tumor's volume was segmented into separate areas, each with consistent signal intensity across sequences (T1WI, T2WI and ADC). For each patient, the signal intensities from all voxels within the ROI and across three imaging modalities were compiled into a comprehensive matrix. This matrix, with dimensions reflecting the voxel count and imaging modalities, was then subjected to voxel-based K-means clustering. This process effectively partitioned the tumor into distinct, spatially unique regions, each with a uniform signal intensity pattern, achieving automated segmentation of these heterogeneous habitats. The Calinski-Harabasz index was applied to determine the most suitable number of clusters, which was tested across a range from two to ten. Using the OnekeyAI platform, we imported each patient's ROI into the platform's components and classified the uterine lesions into three classes named habitat_1, habitat_2, and habitat_3. We applied a minimum subregion size of 64 voxels (4×4×4 mm³ at our resampled resolution), which we considered sufficient for stable calculation of matrix-based texture features. None of the lesions had habitat subregions falling below this threshold after clustering.

### Feature Selection and Model Development

**Figure 2:**
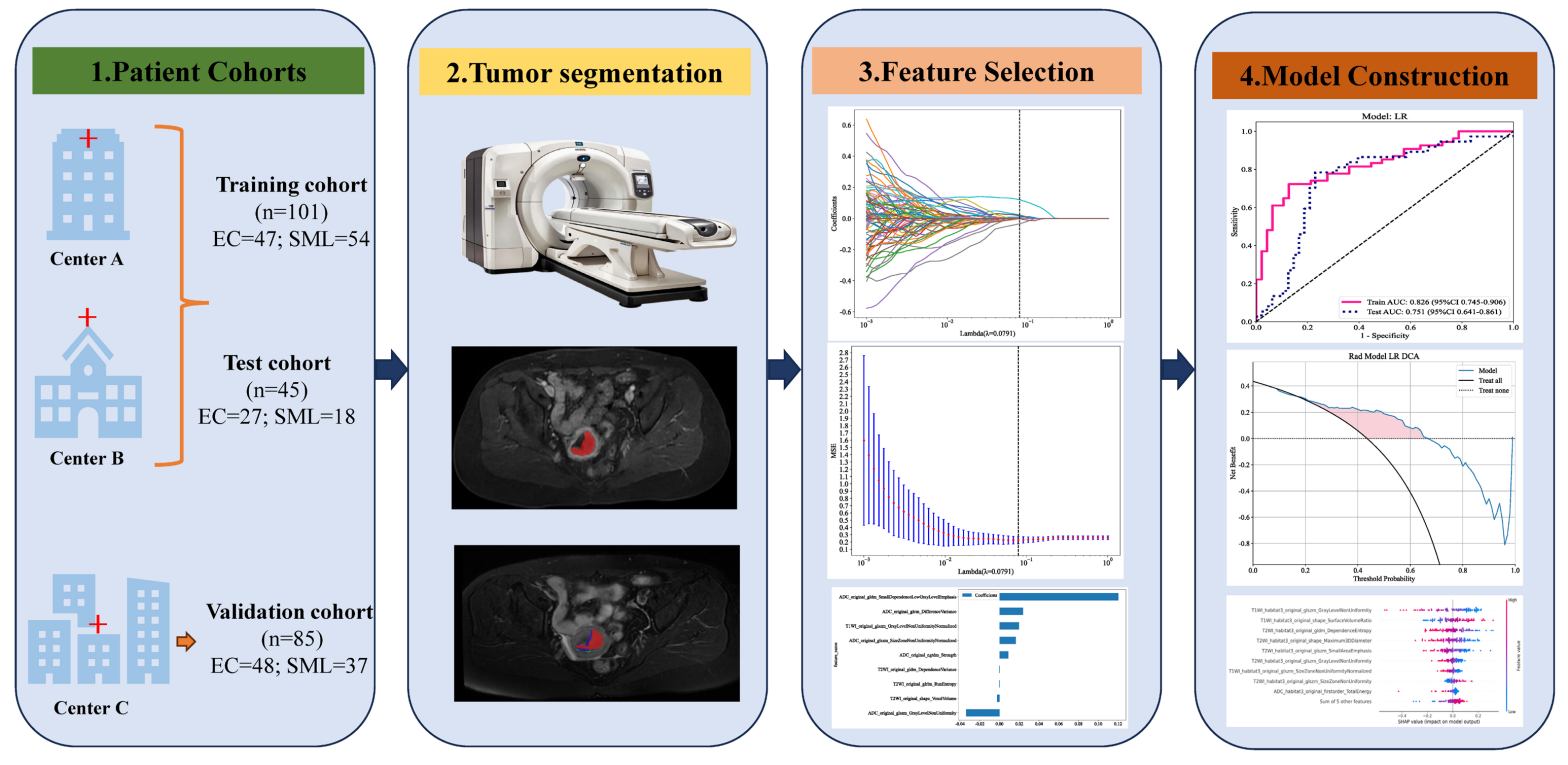
The overall workflow of this study.

After segmenting the ROI, radiomic feature analysis was performed by PyRadiomics that enabled the extraction of first-order statistics, shape features, gray-level co-occurrence matrix (GLCM), gray-level dependence matrix (GLDM), gray-level run-length matrix (GLRLM), gray-level size-zone matrix (GLSZM), and neighborhood gray-tone difference matrix (NGTDM), totaling 107 features per image sequence. Radiomic features were extracted from the entire ROI and each sub-region. All features were standardized using Z-score normalization to ensure a normal distribution. During the feature reduction process, various methods were applied, including t-tests, Pearson correlation analysis, and the mRMR algorithm. The selection of the most discriminative features was further refined using the Least Absolute Shrinkage and Selection Operator (LASSO) algorithm, with the stability of the feature selection confirmed through 10-fold cross-validation. A logistic regression (LR) classification model was developed in the training cohort based on features extracted from each habitat and the whole-tumor with five-fold cross-validation and finally validated in test cohort. Decision curve analysis (DCA) was conducted to assess the clinical applicability of the models. Calibration curves were generated to illustrate the calibration performance of the models in both the training and validation cohorts. The shapley additive explanations (SHAP) algorithm was used to visually show each feature's contribution to the predictions, enhancing model interpretability. **Figure 2** exhibits the overall workflow of this study.

### Statistical Analysis

The statistical analysis was performed using SPSS 24.0 (IBM, USA) and Python 3.11.4 with key packages (PyRadiomics v3.0.1, scikit-learn v1.3.0, scipy v1.11.1, numpy v1.24.3, pandas v2.0.3, matplotlib v3.7.1, shap v0.42.1). In our analysis, continuous variables were compared using the Student's t-test and the Mann-Whitney U test. For categorical data, we utilized the Chi-square test. Model efficacy was assessed by analyzing the area under the receiver operating characteristic (ROC) curve. The DeLong test was applied for the comparative analysis of ROC curves. P<0.05 was considered statistical significance.

## Results

### Patient Characteristics

The clinical features of patients are summarized in **Table 1**. A total of 231 patients (97 EC and 134 SML) were acquired from three centers. 50 cases from center A and 96 cases from center B were randomly divided into training (101 cases) and test (45 cases) sets in a 7:3 ratio, and 85 cases from center C constituting the external validation set.

### Feature Selection

**Figure 3:**
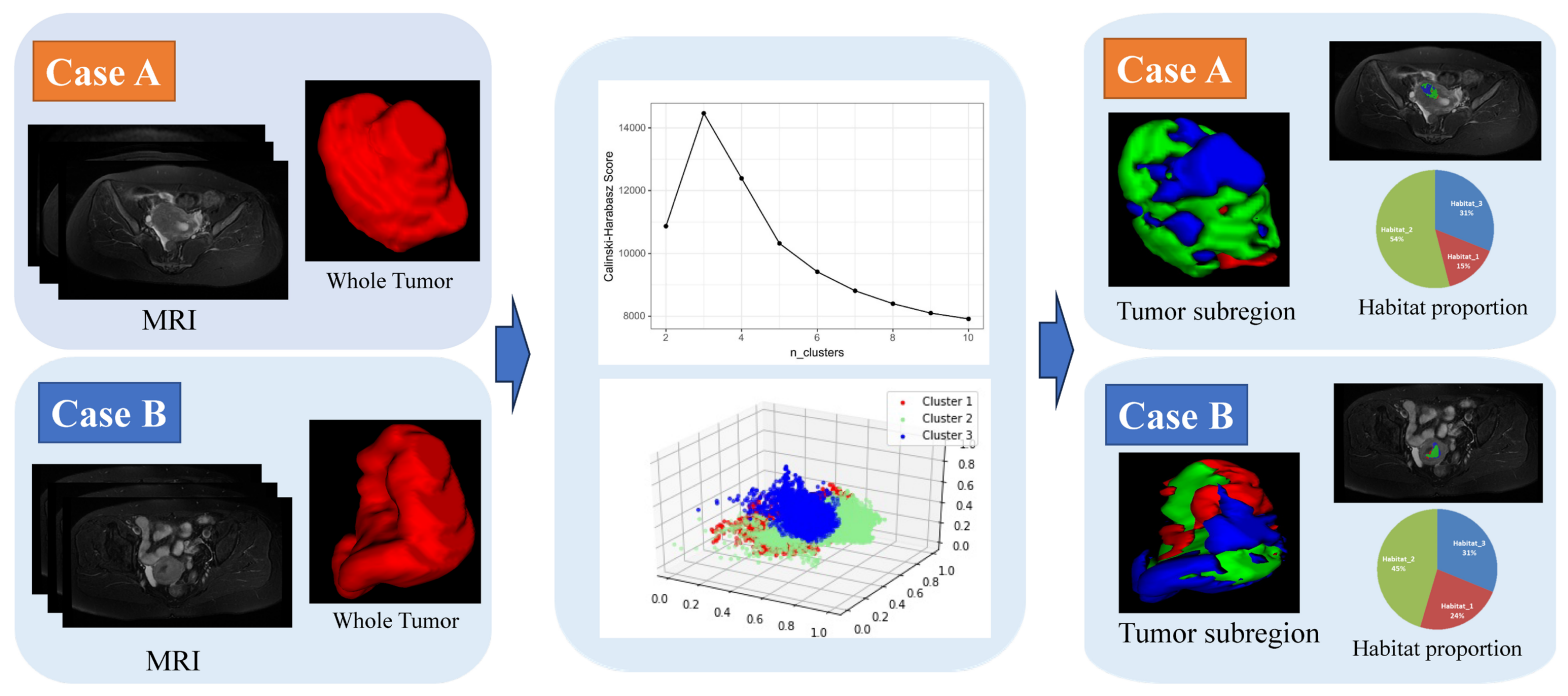
Habitat clustering process. Case A: a submucous leiomyoma patient; Case B: an endometrial carcinoma patient.

The optimal number of clusters was determined to be three based on the Calinski-Harabasz score **(Figure 3)**. A total of 321 features were extracted from the imaging data based on habitat_1, habitat_2, habitat_3, and the whole-tumor. After screening the features using ICC values < 0.75, Pearson correlation coefficients and LASSO regression method for model building, yielding 10, 6, 14, and 9 best features based on habitat_1, habitat_2, habitat_3 and the whole-tumor, respectively. These results are presented in the **Table 2 and Supplementary Materials**.

### Model Performance

**Figure 4:**
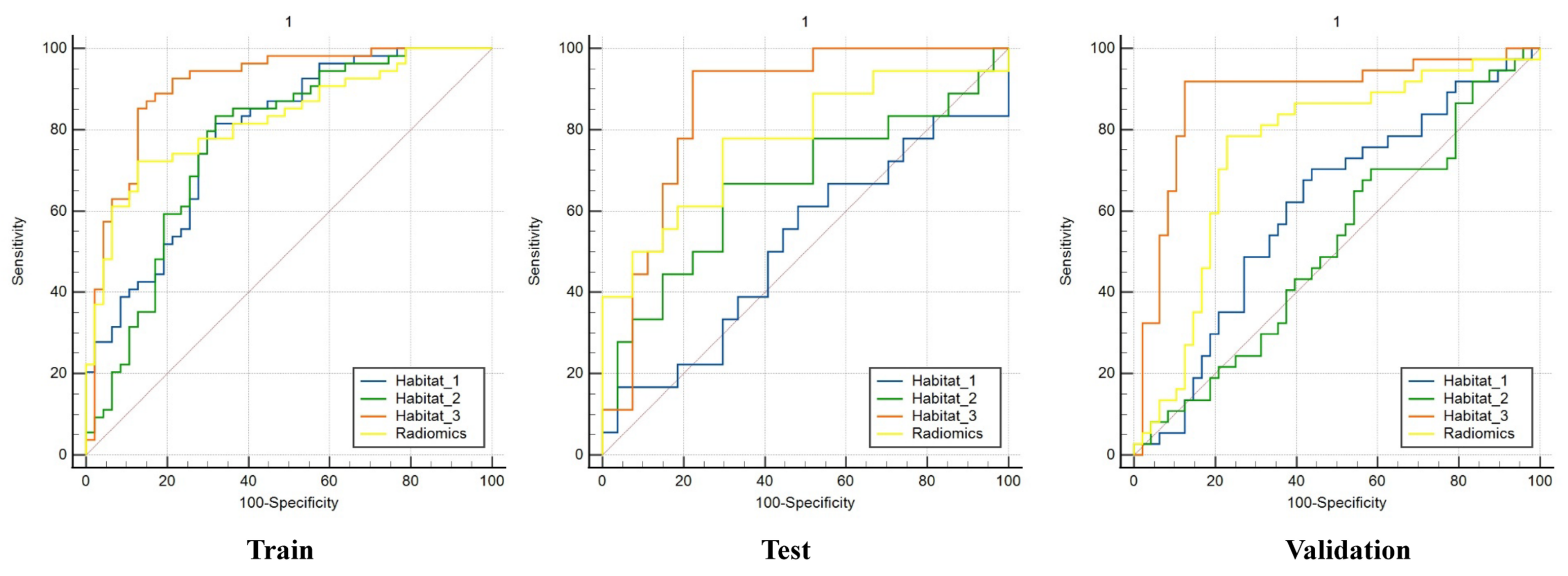
The receiver operating characteristic (ROC) curves of the habitat and radiomics models in the train, test and external validation cohorts.

**Figure 5:**
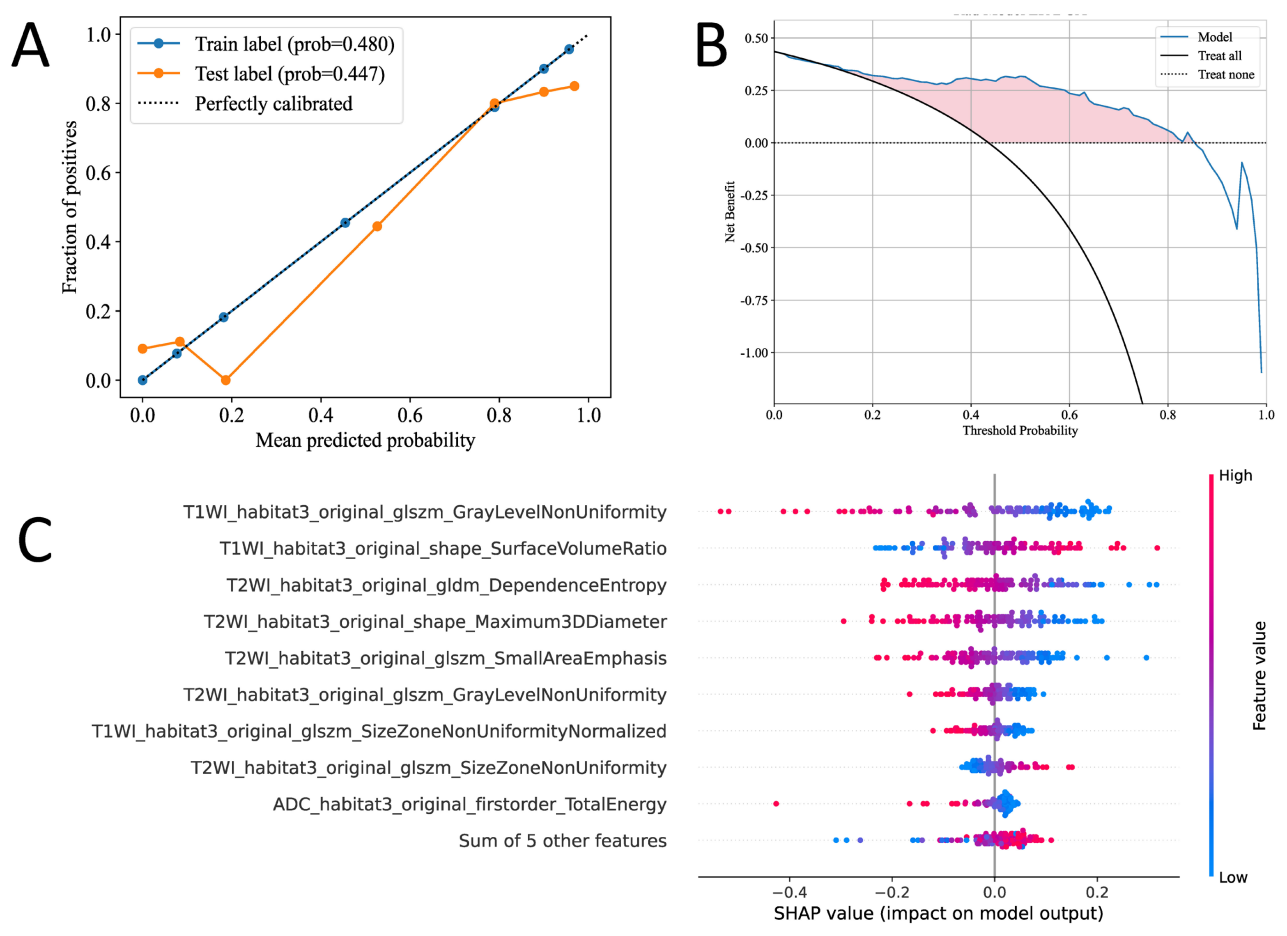
The Calibration curves of habitat_3 model in both the training and validation cohorts. The decision curve analysis (DCA) in habitat_3 model (B). The Shapley additive explanations (SHAP) of each feature in habitat_3 model (C).

We developed LR machine learning models based on the most distinctive imaging histological characteristics of habitat_1, habitat_2, habitat_3, and the whole-tumor. The prediction efficiency of each model is summarized in** Table 3**. **Figure 4** illustrates the receiver operating characteristic curves of the LR machine learning models in training cohort, with area under the curves (AUCs) of 0.787 (95% confidence interval [CI]: 0.699-0.876), 0.770 (95% CI: 0.674-0.865), 0.907 (95% CI: 0.845-0.969), and 0.826 (95% CI: 0.754-0.906) for habitat_1, habitat_2, habitat_3, and the whole-tumor model, respectively. The test cohort had AUCs of 0.486 (95% CI: 0.303-0.668), 0.663 (95% CI: 0.488-0.837), 0.858 (95% CI: 0.745-0.906), and 0.774 (95% CI: 0.624-0.923) for habitat_1, habitat_2, habitat_3, and the whole-tumor model, respectively. The external validation cohort had AUCs of 0.608 (95% CI: 0.486-0.730), 0.514 (95% CI: 0.389-0.638), 0.881 (95% CI: 0.799-0.964), and 0.751 (95% CI: 0.641-0.861) for habitat_1, habitat_2, habitat_3, and the whole-tumor model, respectively. The DeLong test revealed statistically significant differences between habitat_3 and the whole-tumor models in the external validation cohort (p=0.042). The DCA and SHAP summary plot for the habitat_3 model are shown in **Figure 5**. To assess the added clinical value of habitat radiomics beyond standard diagnostic methods, we compared three baseline approaches on the external validation cohort: clinical variables (age, BMI, hypertension, diabetes: AUC 0.597), conventional MRI assessment by two experienced radiologists (AUC 0.679), and mean ADC measurements (threshold 1.29 × 10⁻³ mm²/s: AUC 0.713). Habitat_3 significantly outperformed all baselines (AUC 0.881; DeLong test p < 0.001, p = 0.003, and p = 0.029, respectively), demonstrating incremental diagnostic utility beyond currently available methods.

## Discussion

Distinguishing EC from SML is crucial for the urgent need of personalized treatment in patients with uterine tumors. This study developed more accurate models leveraging intratumoral heterogeneity, which is a key factor in tumor biology and response to therapy. Compared to radiomics models based on the entire tumor, our habitat model demonstrated superior performance in differentiating EC from SML, highlighting the importance of considering tumor heterogeneity in diagnostic models. Among the models evaluated, the habitat_3 model achieved the best performance, indicating significant improvements in model accuracy and predictive gains, which underscores the potential of habitat-based models in enhancing clinical decision-making for uterine tumor management.

Radiomics serves as a pivotal link between medical imaging and personalized medicine, enhancing the precision of cancer diagnostics, prognostics, and predictions through the extensive extraction of quantitative image characteristics from medical imaging data 11. Recent studies have shown that radiomics based on MRI could be used to differentiate EC from other uterine lesions and predict prognosis 12-15. Shen et al. 16 developed MRI-based radiomics models for discriminating EC from endometrial polyps in multicenter. Zhang et al. 17 developed a MRI-based radiomics combining clinical information for distinguishing EC from atypical endometrial hyperplasia. The predictive performance of the individual MRI-based radiomics model is not good enough (AUC = 0.751) in validation cohort. A potential explanation lies in radiomics disregard for the tumor spatial context, which hinders a thorough assessment of its heterogeneity.

Our habitat_3 model (AUC 0.881) outperformed conventional whole-tumor radiomics (AUC 0.751). Unlike deep learning, the habitat approach offers biological interpretability, moderate data requirements, IBSI-compliant features, and clinical transparency via SHAP analysis. Furthermore, habitat_3 demonstrated superior performance compared to clinically available approaches on external validation, including clinical variables alone (AUC 0.597), conventional radiologist assessment (AUC 0.679), and mean ADC measurements (AUC 0.713), establishing clear added value beyond standard diagnostic practice. Tumor heterogeneity within malignant neoplasms is a defining trait, leading to variations in growth velocity, invasiveness, metastatic potential, drug responsiveness, and patient outcomes 7. To precisely quantify the diversity within tumors, a non-invasive imaging method termed habitat imaging has been introduced. This technique aids in the assessment of tumor heterogeneity by categorizing the tumor into various habitats, providing a detailed analysis of the tumor's internal variations. Clustering similar voxels within multi-parameter MRI data, based on a data-driven methodology without any preconceived biases, enables the identification of regions with analogous tissue characteristics on a voxel-by-voxel basisn 18. Contrasting with whole-tumor radiomics, habitat imaging, which concentrates on the omics analysis of tumor sub-regions, delivers a more precise quantification of areas within the tumor that are directly related to its growth and invasive properties^19^. Du et al. 20 identified that clustering metrics, including the Calinski-Harabasz Index, are effective in predicting gene mutations in breast cancer when employed in standalone or multi-metric modeling approaches. Syed et al. 21 revealed a striking congruence between the characteristics observed in imaging and those identified through histological analysis in breast cancer. MRI-based habitat imaging was gradually applied to differential diagnosis in tumor patients. In this study, three habitats were generated based on the Calinski-Harabasz score.

Habitat radiomics synthesizes the strengths of conventional radiomics with the nuances of intratumoral spatial heterogeneity, leading to successful evaluations in cancers such as breast, cervical and ovarian 22-24. Radiomics has not only accurately forecasted cell proliferation levels but also surpassed the performance of traditional radiomics models 25, 26, corroborating the results obtained in our study. The optimal three-habitat solution was objectively determined by the highest Calinski-Harabasz index among tested cluster numbers (k=2-10). The biological interpretation of these habitats reveals distinct pathological processes: habitat_1 (low T1, variable T2, high ADC) encompasses both hyaline degeneration (low T2, fibrosis) and cystic degeneration (high T2, fluid-filled)—two common leiomyoma subtypes with opposing T2 characteristics that create intrahabitat heterogeneity and explain its poor discriminative performance (AUC 0.486-0.608). Habitat_2 (intermediate signals) represents viable tissue with uniform cellularity lacking specific malignant signatures. Notably, habitat_3 (high T1, high T2, low ADC) achieved superior performance (AUC 0.881) by capturing the "malignant triad" of endometrial carcinoma: subacute hemorrhage (high T1), edema/necrosis (high T2), and hypercellularity (low ADC). Unlike leiomyomas exhibiting isolated degenerative patterns, carcinoma demonstrates co-existing hemorrhage, necrosis, and high cellularity; habitat_3 isolates this high-yield signature while excluding the confounding heterogeneity that compromises whole-tumor analysis. Our study observed that the most impactful features contributing to the radiomics model originated from ADC maps, indicating that ADC maps are particularly effective in differentiating EC from SML. Previous studies have confirmed that whole-lesion ADC histogram analysis could serve as an imaging biomarker for differentiating EC from benign endometrial lesions 27-29. The ADC quantifies the Brownian motion of water molecules, with a decrease in ADC values corresponding to higher tumor cellularity, a characteristic commonly observed in malignant lesions 30. Subsequently, habitat_3 model's better performance using T1 and T2 than whole-tumor model using ADC is in contrast to existing studies which primarily employ ADC to differentiate between the two. Malignant or potentially malignant uterine lesions are more prone to exhibit local heterogeneous structures, hemorrhage, and infiltration, which often present as high signal intensities on T1WI and T2WI maps 31, 32. Our study harnessed the combined strengths of T1WI, T2WI, and ADC maps through habitat clustering, facilitating a holistic evaluation of EC and SML, both globally and in sub-regional contexts. Employing habitat, a method for solid tumor segmentation in preoperative imaging, we extracted radiomics from the defined tumor sub-regions. This refined approach systematically omitted regions unrelated to the differential diagnosis of benign versus malignant uterine conditions, enhancing the model's predictive accuracy.

This study had some limitations. Firstly, the test cohort (n=45) and single-center external validation may restrict generalizability. Prospective multi-center studies with larger cohorts are needed to validate the habitat_3 model across diverse settings. Future studies should aim for larger, prospective analyses. To solidify the biological relevance of habitat sub-regions, a direct comparison of pathological tissue with corresponding MRI sub-regions is necessary. Secondly, the extensive size and range of lesions made manual segmentation burdensome, potentially affecting feature stability. Automated segmentation techniques are needed to enhance efficiency and reliability. Thirdly, scanner heterogeneity may have influenced our results. Although N4 correction and isotropic resampling were applied, residual differences in signal and contrast between platforms may still have affected habitat clustering. Further validation across more centers and scanner types is needed. Lastly, our study focused on clustering three standard MRI sequences; further exploration of additional quantitative functional MRI sequences is warranted, such as diffusion kurtosis imaging and dynamic contrast-enhanced MRI.

In this exploratory multicenter study, MRI-based habitat radiomics, particularly the habitat_3 model, showed promise for differentiating early-stage EC from SML, with incremental improvement over conventional whole-tumor radiomics. However, prospective validation in larger, diverse cohorts is required before clinical application.

## Supplementary Material

Supplementary figures and tables.

## Figures and Tables

**Table 1:** Baseline clinical characteristics of the patients.

Features	Training cohort		p-value	Test cohort		p-value	Validation cohort		p-value
	EC	SML		EC	SML		EC	SML	
Age	59.51±10.78	50.70±9.24	<0.001	54.04±11.24	52.00±14.46	0.598	57.13±8.96	50.84±7.21	<0.001
BMI	28.04±4.52	26.19±3.60	0.022	31.56±2.40	30.15±2.73	0.076	26.82±3.92	27.05±3.68	0.972
Hypertension			1			0.176			0.578
Absent	40(85.11%)	47(87.04%)		20(74.07%)	17(94.44%)		41(85.42%)	29(78.38%)	
Present	7(14.89%)	7(12.96%)		7(25.93%)	1(5.56%)		7(14.58%)	8(21.62%)	
Diabetes			0.689			0.114			0.931
Absent	34(72.34%)	36(66.67%)		17(62.96%)	16(88.89%)		34(70.83%)	25(67.57%)	
Present	13(27.66%)	18(33.33%)		10(37.04%)	2(11.11%)		14(29.17%)	12(32.43%)	

EC: endometrial carcinoma; SML: submucous leiomyoma

**Table 2:** Key Radiomic Features Selected for Each Model.

Model	Feature	Category	Sequence	Weight
Habitat_1	T2WI_glszm_SizeZoneNonUniformity	Texture	T2WI	-0.048
	T2WI_shape_Maximum3DDiameter	Shape	T2WI	-0.040
	T1WI_glszm_LowGrayLevelZoneEmphasis	Texture	T1WI	0.027
	ADC_ glszm_ ZonePercentage	Texture	ADC	0.016
Habitat_2	ADC_ glrlm_ ShortRunEmphasis	Texture	ADC	0.055
	T2WI_shape_Maximum3DDiameter	Shape	T2WI	-0.043
	T2WI_glszm_SizeZoneNonUniformity	Texture	T2WI	-0.037
Habitat_3	T2WI_shape_Maximum3DDiameter	Shape	T2WI	-0.112
	T1WI_glszm_GrayLevelNonUniformity	Texture	T1WI	-0.128
	T1WI_shape_SurfaceVolumeRatio	Shape	T1WI	0.06
	ADC_glcm_Imc1	Texture	ADC	0.029
Whole-tumor	ADC_gldm_SmallDependenceLowGrayLevelEmphasis	Texture	ADC	0.12
	ADC_glszm_GrayLevelNonUniformity	Texture	ADC	-0.034

Note: Only features with |coefficient| > 0.02 shown for brevity. Full feature lists in Supplementary Material. GLCM: gray-level co-occurrence matrix; GLRLM: gray-level run-length matrix; GLSZM: gray-level size-zone matrix; GLDM: gray-level dependence matrix.

**Table 3:** Performance of each model.

Models		AUC (95% CI)	Accuracy (95% CI)	Sensitivity (95% CI)	Specificity (95% CI)	PPV (95% CI)	NPV (95% CI)
Training cohort	Habitat_1	0.787 (0.699-0.876)	0.752 (0.660-0.826)	0.815 (0.692-0.896)	0.681 (0.538-0.796)	0.746 (0.622-0.839)	0.762 (0.615-0.865)
	Habitat_2	0.770 (0.674-0.865)	0.762 (0.671-0.835)	0.833 (0.713-0.910)	0.681 (0.538-0.796)	0.750 (0.628-0.842)	0.780 (0.633-0.880)
	Habitat_3	0.907 (0.845-0.969)	0.861 (0.781-0.916)	0.852 (0.734-0.923)	0.872 (0.748-0.940)	0.885 (0.770-0.946)	0.837 (0.710-0.915)
	Radiomics	0.826 (0.745-0.906)	0.792 (0.703-0.860)	0.722 (0.591-0.824)	0.872 (0.748-0.940)	0.867 (0.738-0.937)	0.732 (0.604-0.830)
Test cohort	Habitat_1	0.486 (0.303-0.668)	0.667 (0.521-0.786)	0.167 (0.058-0.392)	1.000 (0.875-1.000)	1.000 (0.439-1.000)	0.643 (0.492-0.770)
	Habitat_2	0.663 (0.488-0.837)	0.689 (0.543-0.805)	0.667 (0.437-0.837)	0.704 (0.515-0.841)	0.600 (0.387-0.781)	0.760 (0.566-0.885)
	Habitat_3	0.858 (0.745-0.971)	0.844 (0.712-0.923)	0.944 (0.742-0.990)	0.778 (0.592-0.894)	0.739 (0.535-0.875)	0.955 (0.782-0.992)
	Radiomics	0.774 (0.624-0.923)	0.733 (0.590-0.840)	0.778 (0.548-0.910)	0.704 (0.515-0.841)	0.636 (0.430-0.803)	0.826 (0.629-0.930)
Validation cohort	Habitat_1	0.608 (0.486-0.730)	0.624 (0.517-0.719)	0.703 (0.542-0.825)	0.562 (0.423-0.693)	0.553 (0.412-0.686)	0.711 (0.552-0.830)
	Habitat_2	0.514 (0.389-0.638)	0.541 (0.436-0.643)	0.703 (0.542-0.825)	0.417 (0.288-0.557)	0.481 (0.354-0.611)	0.645 (0.469-0.789)
	Habitat_3	0.881 (0.799-0.964)	0.894 (0.811-0.943)	0.919 (0.787-0.972)	0.875 (0.753-0.941)	0.850 (0.709-0.929)	0.933 (0.821-0.977)
	Radiomics	0.751 (0.641-0.861)	0.776 (0.677-0.852)	0.784 (0.628-0.886)	0.771 (0.635-0.867)	0.725 (0.572-0.839)	0.822 (0.687-0.907)

AUC: area under the receiver operating characteristic curve; PPV: positive predictive values; NPV: negative predictive values.
