# Supplementary material for: MRI-Based Habitat Radiomics for Differentiating Early-Stage Endometrial Carcinoma from Submucous Leiomyoma: A Multicenter Validation Study
Source: J Cancer. 2026 May 18;17(5):1073–81. doi: 10.7150/jca.129931 (PMC13189845; doi:10.7150/jca.129931)
Supplement: Supplementary file 1 — Supplementary figures and tables. [file jcav17p1073s1.pdf]

Supplemental material

Habitat_1

Rad-score = 0.5346534653465347 + +0.002466 * ADC_habitat1_original_firstorder_Median +0.004711 * ADC_habitat1_original_glcm_DifferenceAverage +0.012096 * ADC_habitat1_original_glrlm_RunEntropy +0.016264 * ADC_habitat1_original_glszm_ZonePercentage -0.016026 * T1WI_habitat1_original_glcm_MCC +0.026693 * T1WI_habitat1_original_glszm_LowGrayLevelZoneEmphasis -0.013995 * T1WI_habitat1_original_glszm_SmallAreaEmphasis -0.047753 * T2WI_habitat1_original_glszm_SizeZoneNonUniformity -0.001059 * T2WI_habitat1_original_ngtdm_Strength -0.040232 * T2WI_habitat1_original_shape_Maximum3DDiameter

Habitat_2

Rad-score = 0.5346534653465347 + +0.003455 * ADC_habitat2_original_gldm_SmallDependenceLowGrayLevelEmphasis -0.000251 * ADC_habitat2_original_glrlm_GrayLevelNonUniformity +0.055132 * ADC_habitat2_original_glrlm_ShortRunEmphasis +0.029268 * ADC_habitat2_original_glszm_ZonePercentage -0.036839 * T2WI_habitat2_original_glszm_SizeZoneNonUniformity -0.042679 *

T2WI_habitat2_original_shape_Maximum3DDiameter

Habitat_3

Rad-score = 0.5346534653465348 -0.017858 * ADC_habitat3_original_firstorder_TotalEnergy +0.028537 * ADC_habitat3_original_glcm_Imc1 +0.000651 * ADC_habitat3_original_glcm_InverseVariance -0.005134 * ADC_habitat3_original_glszm_SizeZoneNonUniformity -0.000752 * ADC_habitat3_original_glszm_ZoneVariance -0.024819 * T1WI_habitat3_original_glrlm_LongRunHighGrayLevelEmphasis -0.127622 * T1WI_habitat3_original_glszm_GrayLevelNonUniformity -0.034406 * T1WI_habitat3_original_glszm_SizeZoneNonUniformityNormalized +0.060308 * T1WI_habitat3_original_shape_SurfaceVolumeRatio -0.038740 * T2WI_habitat3_original_gldm_DependenceEntropy -0.003480 * T2WI_habitat3_original_glszm_GrayLevelNonUniformity -0.012685 * T2WI_habitat3_original_glszm_SizeZoneNonUniformity -0.031650 * T2WI_habitat3_original_glszm_SmallAreaEmphasis -0.111754 * T2WI_habitat3_original_shape_Maximum3Ddiameter

Radiomics

Rad-score = 0.5346534653465347 + +0.023670 * ADC_original_glcm_DifferenceVariance +0.120254 *

ADC_original_gldm_SmallDependenceLowGrayLevelEmphasis -0.033791 *

ADC_original_glszm_GrayLevelNonUniformity +0.016473 *

ADC_original_glszm_SizeZoneNonUniformityNormalized +0.008790 *

ADC_original_ngtdm_Strength +0.019509 *

T1WI_original_glszm_GrayLevelNonUniformityNormalized -0.000261 *

T2WI_original_gldm_DependenceVariance -0.000373 *

T2WI_original_glrlm_RunEntropy -0.002473 * T2WI_original_shape_VoxelVolume

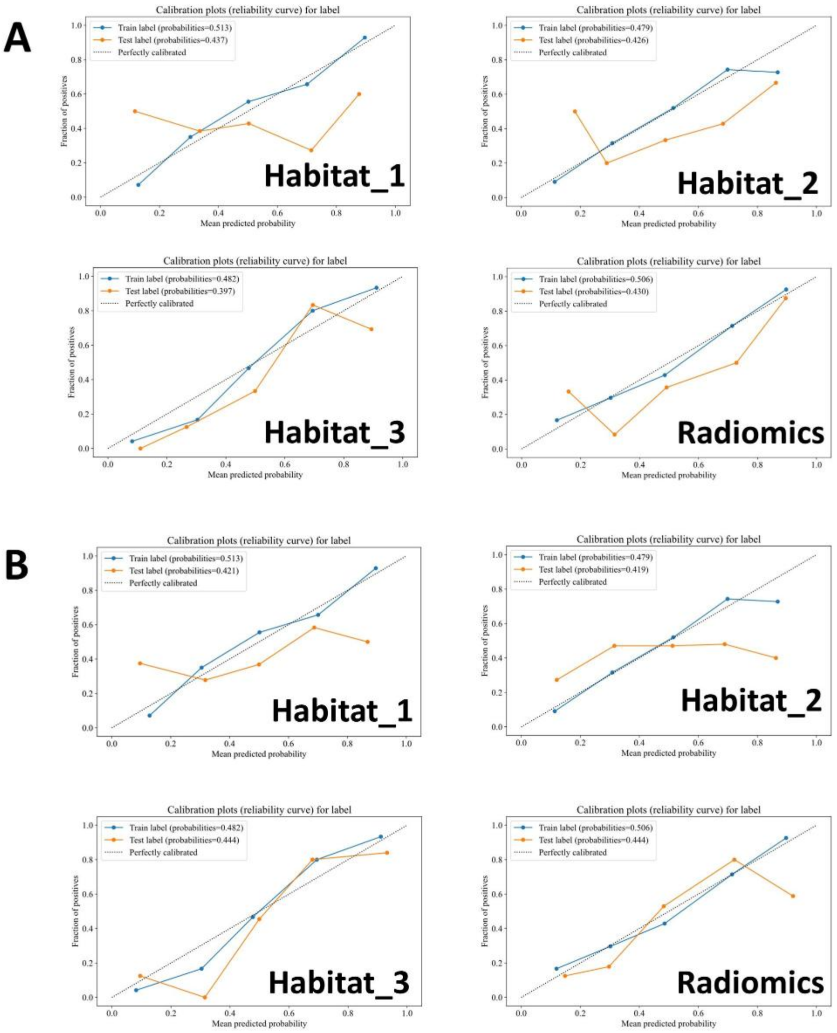

Figure S1. The calibration curves for all habitat models in Test (A) and Validation (B) cohorts.
